# Vascular endothelial overexpression of human CYP2J2 (Tie2-CYP2J2 Tr) modulates cardiac oxylipin profiles and enhances coronary reactive hyperemia in mice

**DOI:** 10.1371/journal.pone.0174137

**Published:** 2017-03-22

**Authors:** Ahmad Hanif, Matthew L. Edin, Darryl C. Zeldin, Christophe Morisseau, John R. Falck, Mohammed A. Nayeem

**Affiliations:** 1 Basic Pharmaceutical Sciences, School of Pharmacy, Center for Basic and Translational Stroke Research. West Virginia University, Morgantown, West Virginia, United States of America; 2 Division of Intramural Research, NIEHS/NIH, Research Triangle Park, North Carolina, United States of America; 3 University of California Davis, Davis, California, United States of America; 4 Biochemistry, University of Texas Southwestern Medical Center, Dallas, Texas, United States of America; Emory University, UNITED STATES

## Abstract

Arachidonic acid is metabolized to epoxyeicosatrienoic acids (EETs) by cytochrome (CYP) P450 epoxygenases, and to ω-terminal hydroxyeicosatetraenoic acids (HETEs) by ω-hydroxylases. EETs and HETEs often have opposite biologic effects; EETs are vasodilatory and protect against ischemia/reperfusion injury, while ω-terminal HETEs are vasoconstrictive and cause vascular dysfunction. Other oxylipins, such as epoxyoctadecaenoic acids (EpOMEs), hydroxyoctadecadienoic acids (HODEs), and prostanoids also have varied vascular effects. Post-ischemic vasodilation in the heart, known as coronary reactive hyperemia (CRH), protects against potential damage to the heart muscle caused by ischemia. The relationship among CRH response to ischemia, in mice with altered levels of CYP2J epoxygenases has not yet been investigated. Therefore, we evaluated the effect of endothelial overexpression of the human cytochrome P450 epoxygenase CYP2J2 in mice (Tie2-CYP2J2 Tr) on oxylipin profiles and CRH. Additionally, we evaluated the effect of pharmacologic inhibition of CYP-epoxygenases and inhibition of ω-hydroxylases on CRH. We hypothesized that CRH would be enhanced in isolated mouse hearts with vascular endothelial overexpression of human CYP2J2 through modulation of oxylipin profiles. Similarly, we expected that inhibition of CYP-epoxygenases would reduce CRH, whereas inhibition of ω-hydroxylases would enhance CRH. Compared to WT mice, Tie2-CYP2J2 Tr mice had enhanced CRH, including repayment volume, repayment duration, and repayment/debt ratio (*P* < 0.05). Similarly, inhibition of ω-hydroxylases increased repayment volume and repayment duration, in Tie2-CYP2J2 Tr compared to WT mice (*P* < 0.05). Endothelial overexpression of CYP2J2 significantly changed oxylipin profiles, including increased EETs (*P* < 0.05), increased EpOMEs (*P* < 0.05), and decreased 8-iso-PGF_2α_ (*P* < 0.05). Inhibition of CYP epoxygenases with MS-PPOH attenuated CRH (*P* < 0.05). Ischemia caused a decrease in mid-chain HETEs (5-, 11-, 12-, 15-HETEs *P* < 0.05) and HODEs (*P* < 0.05). These data demonstrate that vascular endothelial overexpression of CYP2J2, through changing the oxylipin profiles, enhances CRH. Inhibition of CYP epoxygenases decreases CRH, whereas inhibition of ω-hydroxylases enhances CRH.

## Introduction

Arachidonic acid (AA) can be metabolized to epoxyeicosatrienoic acids (EETs) by cytochrome P450 (CYP) epoxygenases, primarily of the CYP2C and CYP2J subfamilies. In a parallel pathway, CYP ω-hydroxylases, such as CYP4A, hydroxylate AA to ω-terminal HETEs (hydroxyeicosatetraenoic acids), including the potent vasoconstrictor 20-HETE [1]. CYP epoxygenases can generate four distinct EET regioisomers: 5,6-, 8,9-, 11,12- and 14,15-EET. EETs are involved in numerous biological functions, including hyperpolarization and relaxation of vascular smooth muscle cells [2, 3]. Mouse cardiomyocytes with increased EET generation are protected against ischemia/reperfusion injury [4, 5]. EETs have short half lives, mainly due to their conversion to dihydroxyeicosatrienoic acids (DHETs) [6] by soluble epoxide hydrolase (sEH). Different strategies have been used to experimentally overcome this shortcoming to assess the beneficial effects of EETs, including endothelial overexpression of CYP epoxygenases, such as CYP2J2 [7, 8]. While humans have just one CYP2J enzyme, CYP2J2, mice express 7 functional CYP2J proteins [9]. CYP2J2 is expressed in different vascular tissues, including the heart muscle and coronary arteries [4, 9]. Isolated aortic endothelial cells from Tie2-CYP2J2 Tr mice had 30% higher production of 11,12- and 14,15-EETs into culture medium [8]. In male mice, endothelial overexpression of human CYP2J2 (Tie2-CYP2J2 Tr) enhanced blood flow and suppressed inflammation to protect against experimental cerebral ischemia [8].

Ischemic insult to the heart is followed by transient increase in coronary blood flow [10] which is known as coronary reactive hyperemia (CRH) [10, 11]. The increased perfusion associated with CRH is protective by increasing nutrient and oxygen supply to the deprived heart muscle and removing metabolic byproducts. Decreased CRH is documented in some pathologic conditions, such as cardiac hypertrophy [12], metabolic syndrome [13], unstable angina, myocardial infarction, and congestive heart failure [14]. Several metabolic mediators regulate CRH, including adenosine [12, 15, 16], nitric oxide (NO) [12], and hydrogen peroxide (H_2_O_2_) [12]. Oxylipins, such as EETs, DHETs, EpOMEs, DiHOMEs, mid-chain HETEs, prostanoids, and HODEs, have been investigated for their vascular effects [7, 17–20]. Oxylipins may play a role in CRH [17].

In addition to EETs, CYP2J2 generates epoxyoctadecaenoic acid (EpOMEs) oxylipins from linoleic acid [21]. EpOMEs protect against hypoxia/reoxygenation injury at physiological levels [20, 22]. Changes in one oxylipin group may coincide with changes in other groups, such as HETEs, hydroxyoctadecadienoic acids (HODEs), and prostanoids [17]. Epidemiological studies suggest a protective role of CYP2J2 against cardiovascular disease in humans [23, 24]. The CYP2J2*7 polymorphism, which is associated with reduced CYP2J2 expression and activity, is linked to a higher risk of adverse cardiovascular events including myocardial infarction [23, 24]. The effects of human CYP2J2 vascular overexpression and the associated changes in oxylipin profiles, and inhibition of CYP-epoxygenases, ω-hydroxylases on CRH in response to short ischemia have not been investigated. We hypothesized that vascular endothelial overexpression of human CYP2J2 enhances CRH through modulation of oxylipin profiles; and inhibition of ω-hydroxylases enhances CRH, whereas inhibition of CYP-epoxygenases reduces CRH.

## Materials and methods

### Animals

The generation of transgenic mice expressing Tie2-driven human 2J2 in endothelial cells on a C57BL/6 genetic background (Tie2-CYP2J2 Tr) was described by Lee et al. [7, 25]. Tie2-CYP2J2 Tr and wild type (WT) mice were of the C57BL/6 genetic background, and were generously provided by Dr. Darryl Zeldin, National Institute of Environmental Health Sciences/National Institutes of Health (NIH). All animal care and experimentation protocols were approved and carried out in accordance with the West Virginia University Institutional Animal Care and Use Committee and were in accordance with the principles and guidelines of the NIH’s *Guide for the Care and Use of Laboratory Animals*. Both male and female mice (14–16 wks old) in an equal ratio were used in our study. Mice were maintained in cages with a 12:12 hour light-dark cycle and free access to standard chow (Cat #2018, Envigo, Indianapolis, IN) and water. Diet 2018 contains 6.2% fat by weight, including 0.7% palmitic, 0.2% stearic, 1.2% oleic %, 3.1% linoleic, and 0.3% linolenic Acids.

### Langendorff-perfused heart preparation

We used the constant pressure mode of the Langendorff isolated heart perfusion as previously described [17]. Tie2-CYP2J2 Tr and wild-type mice (14–16 wks.) were euthanized with sodium pentobarbital (100 mg/kg body weight intra-peritoneally). Hearts were excised and immediately placed into heparinized (5 U/mL) ice-cold Krebs-Henseleit buffer containing (in mM) 119.0 NaCl, 11.0 glucose, 22.0 NaHCO_3_, 4.7 KCl, 1.2 KH_2_PO_4_, 1.2 MgSO_4_, 2.5 CaCl_2_, 2.0 pyruvate, and 0.5 EDTA. After removal of the lungs and tissue surrounding the heart, the aorta was rapidly cannulated with a 20-gauge, blunt-ended needle and continuously perfused with 37°C buffer continuously bubbled with [95% O_2_]–[5% CO_2_] at a constant perfusion pressure of 80 mmHg. The left atrium was excised, and a water-filled balloon made of plastic wrap was inserted into the left ventricle through the mitral valve. The balloon was connected to a pressure transducer for continuous measurement of left ventricular developed pressure (LVDP) and heart rate (HR). The heart was then immersed in a water-jacketed perfusate bath (37°C) and left to beat spontaneously. Left ventricular diastolic pressure was adjusted to 2–5 mmHg. A flow transducer was installed above the cannulated aorta for continuous measurement of CF with an ultrasonic flow probe (Transonic Systems, Ithaca, NY). Data were acquired using a Power–Lab Chart data acquisition system (AD Instruments, Colorado Springs, CO). Heart function was allowed to stabilize for 30–40 min before initiation of CRH. Only hearts whose CF increased by more than two fold after a 15-second total occlusion were included in the analysis. This was to include only properly functioning hearts that were not damaged during cannulation and baseline perfusion. Hearts with persistent arrhythmias or LVDP <80 mmHg were excluded.

### Coronary reactive hyperemic response

After stabilization for 30–40 minutes, baseline CF, HR, and LVDP were recorded for isolated Tie2-CYP2J2 Tr and WT mice hearts. Hearts were subjected to 15 seconds of total occlusion by closing the valve directly above the cannulated heart to bring forth CRH. After CF returned to pre-CRH baseline levels, post-CRH baseline CF, CF tracing, HR, LVDP, repayment volume (RV), and repayment duration (RD) recordings were analyzed for each isolated heart. Investigational drugs were infused into the aortic perfusion line using a microinjection pump (Harvard Apparatus, Holliston, MA) for 15 minutes, after which another CRH was induced and the same parameters analyzed again. Drugs were infused at a rate equivalent to 1% of CF. The final concentrations, after standardization of dose (0.01, 0.1, 1, & 10 μM) response for the various drugs used in this study were 1 μM for MS-PPOH (methylsulfonyl-propargyloxyphenylhexanamide, CYP-epoxygenases inhibitor) and 1 μM DDMS (dibromo-dodecenyl-methylsulfimide, CYP4A-blocker). These concentrations are equal or lower than used in previous studies: MS-PPOH, 1 μM [17], and DDMS, 1 μM [26].

### LC–MS/MS oxylipin analysis

Levels of oxylipins (5,6-, 8,9-, 11,12- and 14,15-EET; 5,6-, 8,9-, 11,12- and 14,15-DHET, 5-, 8-, 11-, 12- and 15-HETE; 9,10- and 12,13-EpOME; 9,10- and 12,13-DiHOME; 9- and 13-HODE; 6-keto prostaglandin-F_1α_ [6K-PG-F_1α_], PG-F_2α_, thromboxane B_2_ [TxB_2_], PGD_2_, and PGE_2_), and 8-iso-PGF_2α_ were quantified in pre- and post-CRH heart perfusates of Tie2-CYP2J2 Tr and WT mice through liquid chromatography, tandem mass spectroscopy (LC-MS/MS) as described previously [25]. Heart perfusates were collected for 2.5 min after the first 30 min of stabilization and immediately after reperfusion. Hearts were immersed in 5 mL of warm (37°C) Krebs-Henseleit buffer with 5 μL of 10 μM *t*-AUCB to block EET hydrolysis by sEH. Heart perfusates were collected two times before ischemia (baseline) and pooled together as one sample and two times after ischemia and pooled together as another sample for LC-MS/MS analysis. Samples were stored at –80°C until processing. Samples were spiked with 30 ng PGE_2_-d4, 11,12-EET-d8, and 11,12-DHET-d8 (Cayman) as internal standards, mixed with 0.1 vol of 1% acetic acid in 50% methanol, and extracted by serial passage through Oasis HLB C18 3mL columns (Waters, Milford, MA, USA). Columns were washed twice with 0.1% acetic acid in 5% methanol and eluted with methanol into glass tubes containing 6 μL of 30% glycerol in methanol. The methanol was then evaporated under a stream of nitrogen gas, and the dried tubes were frozen and stored at –80°C until analysis. Online liquid chromatography of extracted samples was performed with an Agilent 1200 Series capillary HPLC (Agilent Technologies, Santa Clara, CA, USA). Separations were achieved using a Halo C18 column (2.7 mm, 10062.1 mm; MAC-MOD Analytical, Chadds Ford, PA), which was held at 50°C. Mobile phase A was 85:15:0.1 water: acetonitrile: acetic acid. Mobile phase B was 70:30:0.1 acetonitrile: methanol: acetic acid. Flow rate was 400 μL/min; Gradient elution was used. Mobile phase percentage B and flow rate were varied as follows: 20% B at 0 min, ramp from 0 to 5 min to 40% B, ramp from 5 to 7 min to 55% B, ramp from 7 to 13 min to 64% B. From 13 to 19 min the column was flushed with 100% B at a flow rate of 550 μL/min. Samples were solvated in 50 μl of 30% ethanol. The injection volume was 10 μL. Samples were analyzed in triplicate. Analyses were performed on an MDS Sciex API 3000 equipped with a TurboIonSpray source (Applied Biosystems). Turbo desolvation gas was heated to 425°C at a flow rate of 6 L/min. Negative ion electrospray ionization tandem mass spectrometry with multiple reaction monitoring was used for detection. Analyte quantification was performed using Analyst 1.5.1 software (AB Sciex, Ontario, Canada). Relative response ratios of analytes and respective internal standards were compared to a standard curve of response ratios for each analyte. Lipid standards, which are sensitive to oxidative degradation, were stored in 100% ethanol under argon and used within 1 year of purchase from Cayman Chemical (Detroit, MI).

### Effect of DDMS (ω-hydroxylases-inhibitor) on CRH response

Isolated Tie2-CYP2J2 Tr and WT mice hearts were stabilized for 30–40 min, followed by 15 sec of total occlusion. Recordings of the first CRH (baseline CF, CF tracing, LVDP, HR, RV, and RD) were analyzed for each heart and averaged. DDMS was infused at a final concentration of 1.0 μM for 15 minutes, after which the second CRH was induced. CRHs before and after DDMS infusion were analyzed and compared.

### Effect of MS-PPOH (CYP-epoxygenases inhibitor) on CRH response

We followed the same protocol in the preceding section: MS-PPOH infused at a final concentration of 1.0 μM for 15 minutes, and a second CRH was induced. CRHs before and after MS-PPOH infusion were analyzed and compared.

### Statistical and data analyses

Flow debt (baseline flow rate multiplied by occlusion duration) and repayment volume (RV; the integral of hyperemic flow above the baseline flow) were calculated using “the integral relative to baseline” function in the data pad of Lab-Chart 7.0 software. Since absolute coronary flow rates change proportionally with heart mass, the RV and flow debt are presented as ml/g wet heart weight, and baseline and peak flow rate data are presented as (mL.min^–1^.g wet heart weight^–1^). Repayment duration is the time period from reperfusion until the perfusion rate returns to the pre-ischemia baseline level. Repayment/debt ratio is the quotient of the repayment volume to flow debt. Values are means ± standard error; *n* represents the number of animals. For data analysis, two-tailed unpaired *t*-test was used for unpaired data analyses, and two-way ANOVA was used to compare data between groups. Differences were considered statistically significant when *P* < 0.05.

## Results

### CRH response

#### Effect of endothelial overexpression of CYP2J2 (Tie2-CYP2J2 Tr) on CRH response

**Fig 1:**
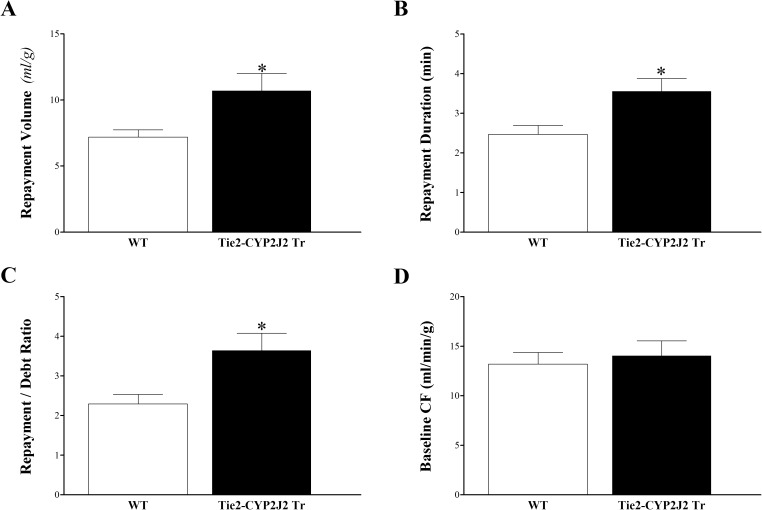
Comparison of coronary reactive hyperemia (CRH) between Tie2-CYP2J2 Tr and WT mice. Repayment volume (A), repayment duration (B), and repayment/debt ratio (C), were increased in Tie2-CYP2J2 Tr compared to WT mice (*P* < 0.05). Baseline CF (D) was not different between the two groups. * *P* < 0.05 versus WT. *n* = 8 per group.

Endothelial overexpression of CYP2J2 enhanced CRH in Tie2-CYP2J2 Tr compared to WT mice. Compared to WT mice, Tie2-CYP2J2 Tr mice had increased repayment volume (7.2 ± 0.5 and 10.7 ± 1.3 mL/g, respectively; *P* < 0.05, **Fig 1A**), increased repayment duration (2.5 ± 0.2 and 3.6 ± 0.3 min, respectively; *P* < 0.05; **Fig 1B**), and increased repayment/debt ratio (2.3 ± 0.2 and 3.6 ± 0.4, respectively; *P* < 0.05; **Fig 1C**). Baseline CF (**Fig 1D**), LVPD, and HR (not shown) were not different between these two groups (*P* > 0.05).

### EETs’ profile analysis of heart perfusate

**Fig 2:**
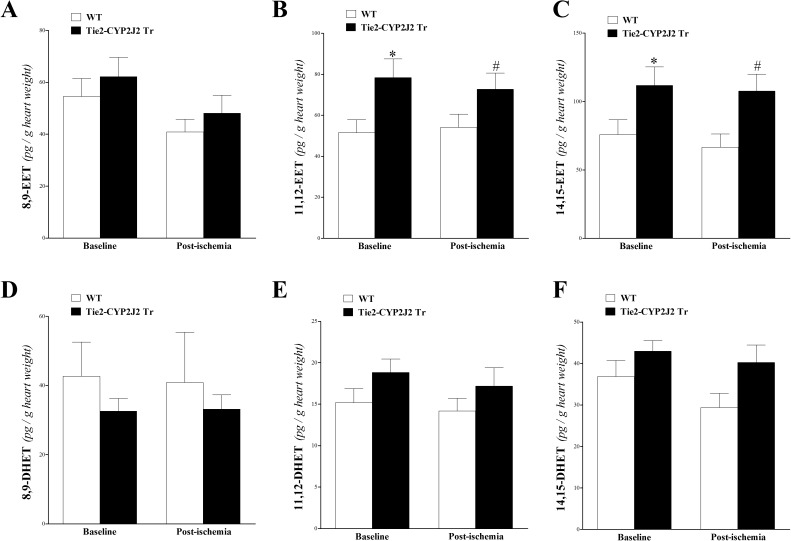
LC–MS/MS analysis for EETs’ (8, 9–, 11, 12–, and 14, 15–) and DHETs’ (8, 9–, 11, 12–, and 14, 15–) levels in WT and Tie2-CYP2J2 Tr mouse heart perfusates at baseline and post-ischemia. 11,12-EET (B), and 14,15-EET (C) were increased in Tie2-CYP2J2 Tr compared to WT mice (*P* < 0.05), whereas 8,9-EET (A) was not significantly different between the two groups (*P* > 0.05). 8,9-DHET (D), 11,12-DHET (E), and 14,15-DHET (F) were not significantly different between WT and Tie2-CYP2J2 Tr mice. Also, the levels of EETs and DHETs were not affected in response to ischemia in either mouse genotype (*P* > 0.05). * *P* < 0.05 versus baseline WT. # *P* < 0.05 versus WT post-ischemia. *n* = 12 per group.

Three out of the four EET regioisomers (8,9-, 11,12-, and 14,15-EETs) and their corresponding metabolites (8,9-, 11,12-, and 14,15-DHETs) were detected by this technique. 11,12-, and 14,15-EETs were increased more in the heart perfusate of Tie2-CYP2J2 Tr vs. WT mice (*P* < 0.05; **Fig 2B and 2C**). 8,9-EET was not different between Tie2-CYP2J2 Tr and WT mice (*P* > 0.05; **Fig 2A**). For DHETs, there was no significant difference between Tie2-CYP2J2 Tr and WT mice (*P* > 0.05; **Fig 2D–2F**). Neither EETs nor DHETs were changed by the short ischemia (*P* > 0.05; **Fig 2A–2F**).

### Effect of MS-PPOH (CYP epoxygenase inhibitor) on CRH response

**Fig 3:**
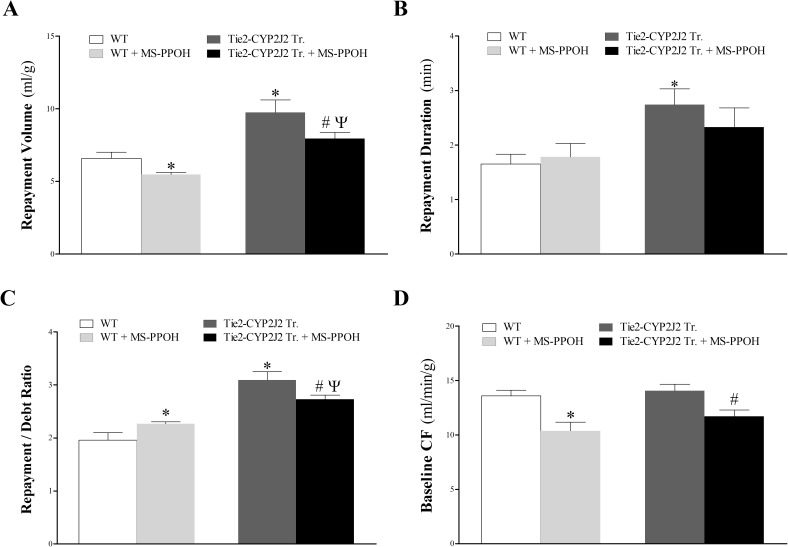
Effect of the CYP-epoxygenases inhibitor, MS-PPOH (1 μM), on coronary reactive hyperemia (CRH) in Tie2-CYP2J2 Tr and WT mice. The CYP-epoxygenases inhibitor, MS-PPOH, decreased CRH in both Tie2-CYP2J2 Tr and WT mice. (A): MS-PPOH decreased repayment volume in both mouse genotypes. However, repayment volume was still higher in untreated Tie2-CYP2J2 Tr vs. untreated WT, and in MS-PPOH-treated Tie2-CYP2J2 Tr vs. MS-PPOH-treated WT. (B): repayment duration was not changed by the treatment with MS-PPOH in both WT and Tie2-CYP2J2 Tr mice, but repayment duration was increased more in untreated Tie2-CYP2J2 Tr vs. untreated WT. (C): MS-PPOH increased repayment/debt ratio in WT mice, but decreased it in Tie2-CYP2J2 Tr mice. Repayment/debt ratio was increased more in untreated Tie2-CYP2J2 Tr vs. untreated WT, and in MS-PPOH-treated Tie2-CYP2J2 Tr vs. MS-PPOH-treated WT. (D): MS-PPOH decreased baseline CF in both mouse genotypes. There was no difference in baseline CF between untreated Tie2-CYP2J2 Tr and untreated WT. * *P* < 0.05 versus untreated WT. # *P* < 0.05 versus untreated Tie2-CYP2J2 Tr mice. ^Ψ^ *P* < 0.05 versus MS-PPOH-treated WT. *n* = 8 per group.

Similar to Fig 1, repayment volume was higher in Tie2-CYP2J2 Tr vs. WT mice. MS-PPOH attenuated CRH in both Tie2-CYP2J2 Tr and WT mice; repayment volume was decreased in WT mice (from 6.6 ± 0.4 to 5.5 ± 0.2 mL/g; *P* < 0.05) and in Tie2-CYP2J2 mice (from 9.8 ± 0.9 to 8.0 ± 0.4 mL/g; *P* < 0.05). Tie2-CYP2J2 Tr hearts repayment volume remained elevated after treating both groups with MS-PPOH (MS-PPOH–treated Tie2-CYP2J2 Tr vs. MS-PPOH–treated WT mice; *P* < 0.05, **Fig 3A**). MS-PPOH also decreased baseline CF in WT mice (from 13.6 ± 0.5 to 10.4 ± 0.8 mL/g; *P* < 0.05) and in Tie2-CYP2J2 Tr mice (from 14.0 ± 0.6 to 11.7 ± 0.6 mL/g; *P* < 0.05, **Fig 3D**). It was not different between the two groups before and after treatment with MS-PPOH (*P* > 0.05, **Fig 3D**). Since the repayment/debt (R/D) ratio is a function of both repayment volume and baseline coronary flow, MS-PPOH affected R/D ratio based on how it affected repayment volume and baseline CF. While MS-PPOH increased R/D ratio in WT mice (from 2.0 ± 0.1 to 2.3 ± 0.6; *P* < 0.05), it decreased it in Tie2-CYP2J2 Tr mice (from 3.1 ± 0.2 to 2.7 ± 0.1; *P* < 0.05, **Fig 3C**). Repayment duration was longer in Tie2-CYP2J2 Tr vs. WT mice (*P* < 0.05; **Fig 3B**), but was not significantly reduced by MS-PPOH (*P* > 0.05; **Fig 3B**). LVPD and HR were not different between and within the two groups (data not shown).

### Mid-chain HETEs’ profile analysis of heart perfusate

**Fig 4:**
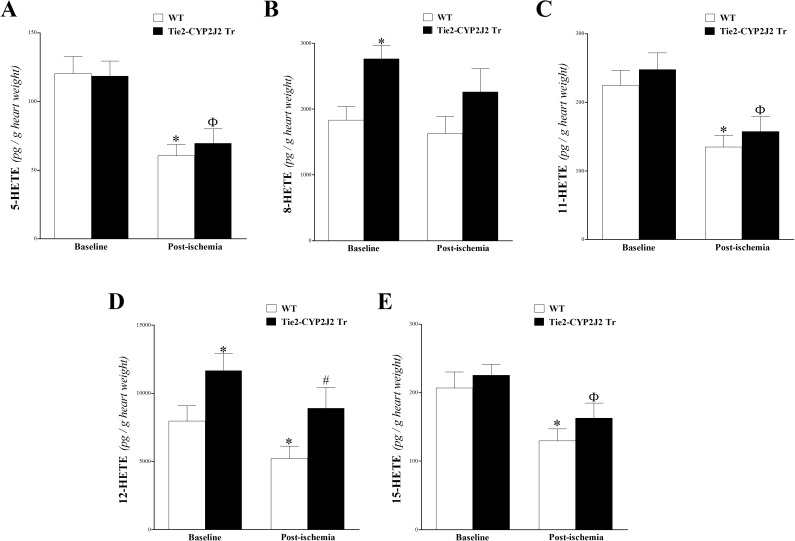
LC–MS/MS analysis of 5-, 8-, 11-, 12- and 15-HETE levels in WT and Tie2-CYP2J2 Tr mouse heart perfusates at baseline and post-ischemia. In Tie2-CYP2J2 Tr mice, the levels of 8-HETE (B) and 12-HETE (D) were increased compared to WT mice at baseline and post-ischemia (*P* < 0.05). In both WT and Tie2-CYP2J2 Tr mice, post-ischemic levels of 5-HETE (A), 8-HETE (B), 11-HETE (C), 12-HETE (D) and 15-HETE (E) were decreased compared to baseline levels (*P* < 0.05). * *P* < 0.05 versus baseline WT. # *P* < 0.05 versus WT post-ischemia. ^Ф^ *P* < 0.05 versus baseline Tie2-CYP2J2 Tr. *n* = 12 per group.

**M**id-chain HETEs (5-, 8-, 11-, 12-, and 15-HETEs) were detected in WT and Tie2-CYP2J2 Tr mouse heart perfusates before and after ischemia. In Tie2-CYP2J2 Tr mice, the levels of 5-, 11-, and 15-HETE were similar those in WT mice at baseline and post-ischemia (*P* > 0.05; **Fig 4A, C and E**, respectively). However, the other detected mid-chain HETEs (8- and 12-HETE) were significantly more elevated at baseline and post-ischemia in Tie2-CYP2J2 Tr compared to WT mice (*P* < 0.05; **Fig 4B and 4D**, respectively). Additionally, the 15-sec ischemia decreased the levels of mid-chain HETEs (reaching significant levels for 5-, 11-, 12-, and 15-HETEs) and in both Tie2-CYP2J2 Tr and WT mice (*P* < 0.05; **Fig 4A–4E**).

**Omega- (ω-) terminal HETEs** (19-, and 20-HETEs) were not detectable in our samples.

### Effect of DDMS (CYP4A inhibitor) on CRH response

**Fig 5:**
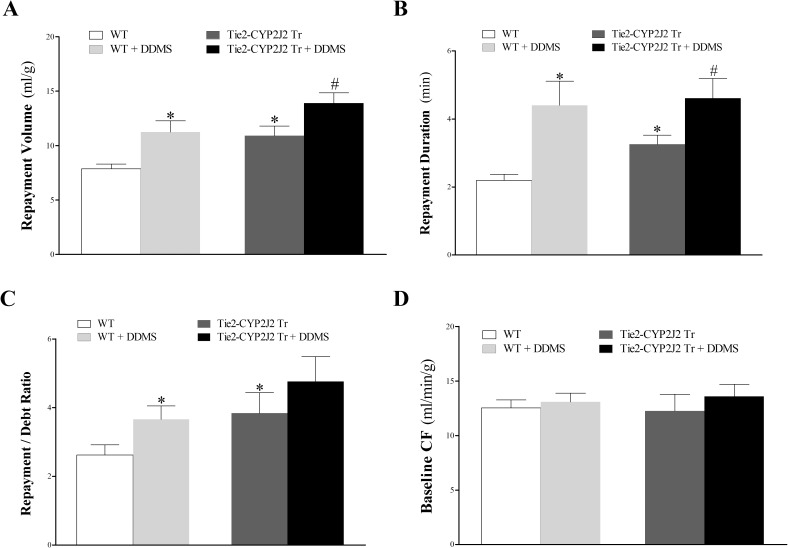
Effect of the CYP4A-blocker, DDMS (1 μM), on coronary reactive hyperemia (CRH) in Tie2-CYP2J2 Tr and WT mice. The CYP4A-blocker, DDMS, enhanced CRH in both Tie2-CYP2J2 Tr and WT mice. Repayment volume (A) and repayment duration (B) were increased by DDMS in both mouse genotypes. Both repayment volume (A) and repayment duration (B) were increased more in untreated Tie2-CYP2J2 Tr compared to untreated WT mice. Similarly, repayment/debt ratio (C) was increased by DDMS in both mouse genotypes, but was significant in WT mice only. Also, repayment/debt ratio was increased more in untreated Tie2-CYP2J2 Tr compared to untreated WT mice. Baseline CF (D) was not different between the two groups. * *P* < 0.05 versus untreated WT. # *P* < 0.05 versus untreated Tie2-CYP2J2 Tr mice. *n* = 8 per group.

DDMS enhanced CRH in both Tie2-CYP2J2 Tr and WT mice. Repayment volume was increased in WT mice (from 7.9 ± 0.5 to 11.3 ± 1.0 mL/g; *P* < 0.05) and in Tie2-CYP2J2 mice (from 10.9 ± 0.9 to 13.9 ± 0.9 mL/g; *P* < 0.05, **Fig 5A**). DDMS also increased repayment duration in WT mice (from 2.2 ± 0.2 to 4.4 ± 0.7 mL/g; *P* < 0.05) and in Tie2-CYP2J2 Tr mice (from 3.3 ± 0.3 to 4.6 ± 0.6 mL/g; *P* < 0.05, **Fig 5B**). Both repayment volume and repayment duration were more increased in Tie2-CYP2J2 Tr vs. WT mice (*P* < 0.05, **Fig 5A and 5B**); however, they were not different between the two groups after infusing DDMS (*P* > 0.05, **Fig 5A and 5B**). Repayment/debt (R/D) ratio had an increasing trend by DDMS in both WT and Tie2-CYP2J2 Tr mice, but was not statistically significant (*P* > 0.05, **Fig 5C**). Baseline CF **(Fig 5D**), LVPD, and HR (data not shown) were not different between and within the two groups (*P* > 0.05).

### Prostanoids’ profile analysis of heart perfusate

**Fig 6:**
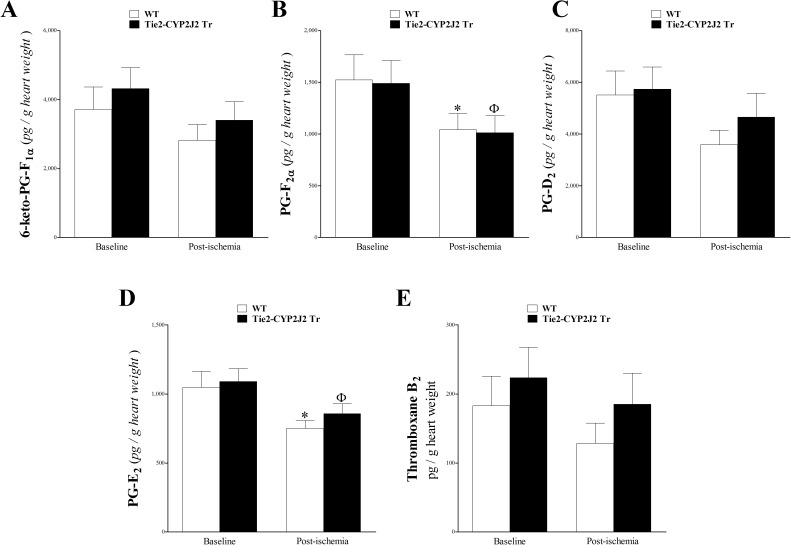
LC–MS/MS analysis of 6-keto-PG-F_1α_, PG-F_2α_, PG-D_2_, PG-E_2_, and TxB_2_ in WT and Tie2-CYP2J2 Tr mouse heart perfusates at baseline and post-ischemia. The levels of prostaglandin 6-Keto-PG-F_1α_ (A), PG-D_2_ and (C), and thromboxane (Tx)-B_2_ (E) were not significantly changed due to ischemia or between Tie2-CYP2J2 Tr and WT mice (*P* > 0.05). In both Tie2-CYP2J2 Tr and WT mice, PG-F_2α_ (B) and PG-E_2_ and (D) were decreased post-ischemia (*P* < 0.05). * *P* < 0.05 versus baseline WT. ^Ф^ *P* < 0.05 versus baseline Tie2-CYP2J2 Tr. *n* = 8 per group.

The levels of 6K-PG-F_1α_, PG-F_2α_, PG-D_2_, PG-E_2_, and TxB_2_ were detected in our LC–MS/MS (**Fig 6**). For these metabolites, no significant difference was noted between WT and Tie2-CYP2J2 Tr mice at baseline or post-ischemia (*P* > 0.05; **Fig 6**). These metabolites generally decreased in response to ischemia in both genotypes, with PG-F_2α_ and PG-E_2_ being significantly reduced (*P* < 0.05; **Fig 6**).

### EpOMEs’ profile analysis of heart perfusate

**Fig 7:**
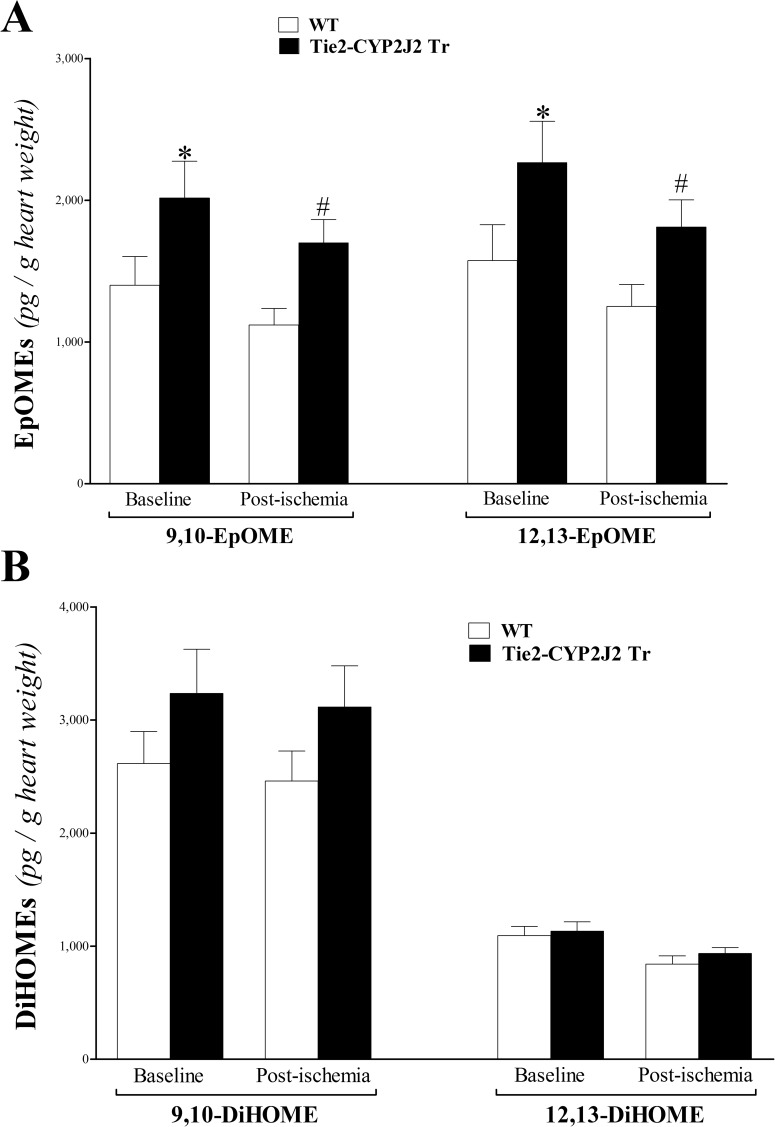
LC–MS/MS analysis of EpOME and DiHOME levels in WT and Tie2-CYP2J2 Tr mouse heart perfusates at baseline and post-ischemia. 9,10- and 12,13-EpOMEs (A) were increased in Tie2-CYP2J2 Tr compared to WT mice at baseline and post-ischemia (*P* < 0.05). 9,10- and 12, 13-DiHOMEs (B) did not change between Tie2-CYP2J2 Tr compared to WT mice (*P* > 0.05). * *P* < 0.05 versus baseline WT. # *P* < 0.05 versus WT post-ischemia. *n* = 8 per group.

Linoleic acid (LA) epoxides (9,10- and 12,13-EpOMEs) were increased in Tie2-CYP2J2 Tr compared to WT mice (*P* < 0.05; **Fig 7A**). In both genotypes, these epoxides had a decreasing trend in response to ischemia that was not statistically significant (*P* > 0.05; **Fig 7A**). The corresponding 9,10- and 12, 13-DiHOME levels were not significantly different between Tie2-CYP2J2 Tr and WT mice (*P* > 0.05; **Fig 7B**).

### HODEs’ profile analysis of heart perfusate

**Fig 8:**
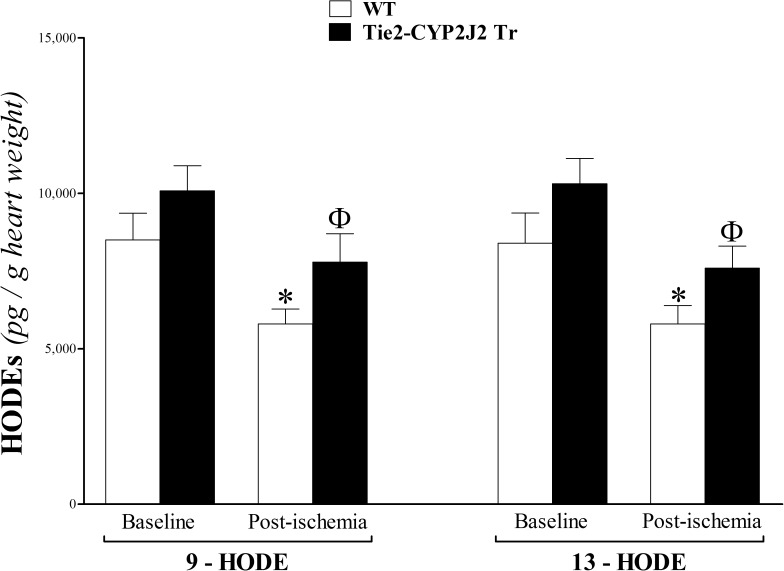
LC–MS/MS analysis of HODEs in WT and Tie2-CYP2J2 Tr mouse heart perfusates at baseline and post-ischemia. In both WT and Tie2-CYP2J2 Tr, 9- and 13-HODEs decreased in response to ischemia (*P* < 0.05). However, they were not significantly different between the two groups at baseline or post-ischemia (*P* > 0.05). * *P* < 0.05 versus baseline WT. ^Ф^ *P* < 0.05 versus WT post-ischemia. *n* = 8 per group.

The hydroxylated LA metabolites, 9- and 13-HODEs, were not significantly different between WT and Tie2-CYP2J2 Tr mice at baseline or post-ischemia (*P* > 0.05; **Fig 8**). However, in both genotypes, 9- and 13-HODEs decreased in response to ischemia (*P* < 0.05; **Fig 8**).

### 8-iso-PGF_2α_ profile analysis of heart perfusate

**Fig 9:**
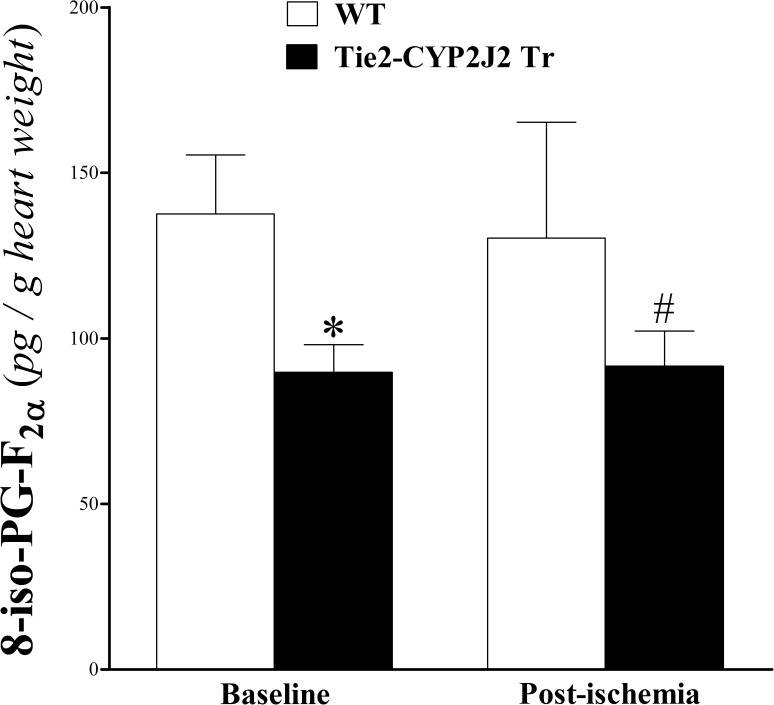
LC–MS/MS analysis of 8-iso-PGF_2α_ in WT and Tie2-CYP2J2 Tr mouse heart perfusates at baseline and post-ischemia. 8-Iso-PGF_2α_ decreased in Tie2-CYP2J2 Tr compared to WT mice at baseline and post-ischemia (*P* < 0.05). However, in both mouse genotypes, 8-Iso-PGF_2α_ was not significantly changed in response to ischemia (*P* > 0.05). *P < 0.05 versus baseline WT. ^#^*P* < 0.05 versus WT post-ischemia. *n* = 8 per group.

**T**his prostaglandin-related isoprostane decreased in Tie2-CYP2J2 Tr versus WT mice at baseline and post-ischemia (*P* < 0.05; **Fig 9**). Brief ischemia did not alter 8-iso-PGF_2α_ in either genotype (*P* > 0.05; **Fig 9**).

**Fig 10:**
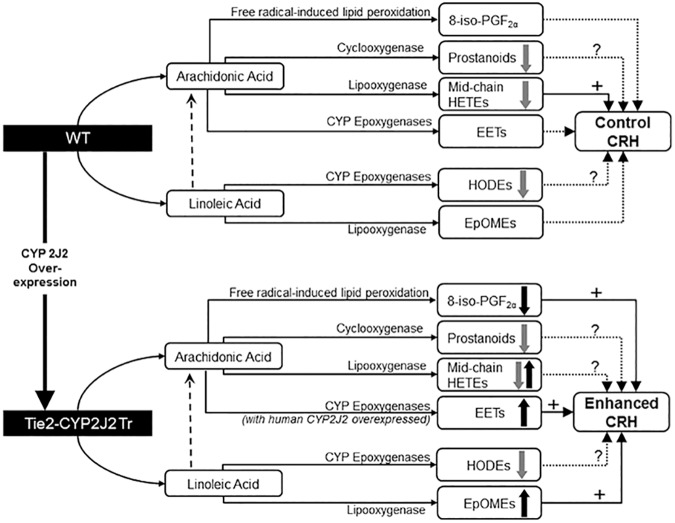
A schematic diagram comparing the oxylipin changes observed in response to both brief ischemia and CYP 2J2 over-expression as well as oxylipins’ possible effect on coronary reactive hyperemia (CRH) in WT and Tie2-CYP2J2 Tr mice. Both ischemia and CYP 2J2 over-expression caused changes in the measured oxylipin profiles. However, the changes associated with ischemia were common to both mouse strains (WT and Tie2-CYP2J2 Tr). Overall, CRH was enhanced in Tie2-CYP2J2 Tr compared to WT possibly through increased EETs, increased EpOMEs, and decreased 8-iso-PGF_2α_. Grey block arrows indicate the effects of ischemia, whereas black block arrows indicate the effects of ischemia CYP 2J2 over-expression.

We summarized our observed results into a proposed schematic diagram (**Fig 10**).

## Discussion

While CYP2J2 overexpression has shown beneficial effects on vasodilation, inflammation, and contractile recovery from cardiac ischemia, the relevance of CYP2J2 metabolism in CRH had not been previously explored. The signaling mechanisms involved in CRH may be substantially different from the mechanisms which improve cardiac function or chronic hypertension in other models. Herein, we investigated the role of endothelial-specific CYP2J2 overexpression and the accompanying oxylipin changes in the modulation of CRH using isolated WT and Tie2-CYP2J2 Tr mouse hearts. Our data demonstrated that: **1)** Endothelial overexpression of CYP2J2 was associated with changes in some oxylipin profiles, including increase in EETs, EpOMEs, and some mid-chain HETEs; **2)** Brief ischemia caused changes in some oxylipin profiles, including decrease in mid-chain HETEs, HODEs, and prostanoids; **3)** Endothelial overexpression of CYP2J2 increased CRH; **4)** Inhibition of CYP epoxygenases (by MS-PPOH) attenuated CRH in WT and Tie2-CYP2J2 Tr mice; and **5)** Inhibition of ω-hydroxylases (by DDMS) enhanced CRH in WT and Tie2-CYP2J2 Tr mice.

Endothelial overexpression of human CYP2J2 increased CRH after brief ischemia in Tie2-CYP2J2 Tr mice compared to WT mice. Ischemia to the heart is likely to cause damage if not corrected within a short time window. The heart responds to ischemia by transiently increasing coronary flow through CRH [10] to reduce the potential for ischemia-induced damage. This protective role of CRH was confirmed by several studies, which indicated that compromised CRH is linked to certain cardiovascular pathologies [12, 13]. We previously demonstrated that the levels of EETs, as well as other oxylipins, correlate with changes in CRH in mice in response to brief ischemia [17]. EETs have well-established beneficial cardiovascular effects [17, 27–31]; they protect against myocardial and cerebral ischemia/reperfusion injury [5, 32], and vasodilate some vascular beds including the intestines [33], kidney preglomerular vasculature [34], and brain [35]. Cytochrome P450 epoxygenase 2J2 (CYP2J2) is expressed in vascular endothelial cells and cardiomyocytes [36], and generates EETs from AA through epoxidation [17]. One of the strategies used experimentally to increase EETs’ levels, in order to study their effects, is the endothelial overexpression of CYP epoxygenases, such as CYP2J2 [7, 8]. Jia et al reported that endothelial overexpression of human CYP2J2 protected against experimental cerebral ischemia in male mice, and that the levels of 11,12- and 14,15-EETs were increased in culture medium containing aortic endothelial cells isolated from the same transgenic mice (Tie2-CYP2J2 Tr) [8]. The authors also suggested that the mechanism of protection was in part linked to enhanced blood flow and suppression of inflammation in Tie2-CYP2J2 Tr mice [8], both of which are recognized effects of EETs [33–35, 37]. In contrast, Tie2-CYP2J2 Tr mice showed no change in vasodilation during reperfusion after 20 minutes, global, no-flow ischemia [7]. In this study, these Tie2-CYP2J2 Tr mice had increased CRH compared to WT mice. The reason for this discrepancy remains unclear. The large vasodilation after 20 minutes of ischemia may be due to release of other factors, such as prostacyclin or nitric oxide, that obscure any contribution of EETs. Our study suggests that CYP2J2-derived EETs do play a significant role in CRH after brief, transient ischemia. Human epidemiological studies also support a protective role of CYP2J2 against cardiovascular disease. The CYP2J2*7 polymorphism, which is associated with reduced CYP2J2 expression and activity, was linked to higher risk of adverse cardiovascular events including myocardial infarction [23, 24]. Correlations with myocardial infarction does little to reveal which processes might be affected in CYP2J2*7 patients; EETs may alter development of atherosclerosis, vasodilation, or protection of myocardium during infarction [38]. Our data do suggest that CYP2J2 expression, correlating with changes in EETs levels, may alter CRH in human subjects with CYP2J2*7 polymorphisms.

We could reverse the effects of epoxygenases using pharmacologic inhibitors. MS-PPOH, a CYP epoxygenase inhibitor, has previously been shown to block the role of EETs as mediators of insulin-mediated augmentation of skeletal muscle perfusion [39]. In our previous study, MS-PPOH significantly reduced CRH in WT mice. We believe this is at least in part due to inhibition of the seven murine CYP2J isoforms [17, 40]. MS-PPOH reduced CRH in Tie2-CYP2J2 Tr mice, though, as reflected by reduced coronary flow of the repayment volume. It remains unclear why coronary flow remained elevated in Tie2-CYP2J2 Tr hearts. Further studies will be needed as we cannot confirm that our dose of MS-PPOH abolished the elevated EETs levels in Tie2-CYP2J2 Tr compared to that in WT.

In addition to EETs, other arachidonic and linoleic acids-derived oxylipins may significantly impact CRH in isolated mouse hearts [17]. Thus, we expanded our oxylipin analyses to examine HETEs, EpOMEs, HODEs, and prostanoids in addition to the EETs. Mid-chain HETEs are produced from AA by lipoxygenase [41] and epoxygenase CYP1B1 [19]. Mid-chain (5-, 8-, 11-, 12- and 15-) HETE levels were affected by two variables in our study: genetic modulation and ischemia. 8- and 12-HETEs were more elevated in Tie2-CYP2J2 Tr compared to WT mice, whereas all measured mid-chain HETEs (5-, 8-, 11-, 12- and 15-HETEs) decreased in response to ischemia in both genotypes. Vasoconstriction and pro-inflammatory effects are among the reported effects of mid-chain HETEs [19, 42]. Also, the increased formation of mid-chain HETEs was involved in cardiovascular dysfunction [43–46]. Our finding that mid-chain HETEs were decreased in both mouse genotypes post-ischemia is in agreement with our previously published data [17]. This finding is not only consistent across the studied mouse genotypes in this and in previous studies [17], but seems intuitive based on the detrimental effects elevated mid-chain HETEs may have on ischemia. However, our finding that 8- and 12-HETEs were more elevated in Tie2-CYP2J2 Tr presents a challenge to the overall observed enhancement of CRH in Tie2-CYP2J2 Tr mice. This finding also contrasts with findings by Jia et al in which 5-, 12- and 15-HETEs were not different between Tie2-CYP2J2 Tr and WT mice [8]. Nonetheless, the different sources of samples may explain this difference: their results were based on cultured aortic endothelial cells [8], whereas ours were based on isolated heart perfusate samples. Therefore, the increased levels of 8- and 12-HETEs may have mitigated the enhanced CRH in Tie2-CYP2J2 Tr mice driven by increased EETs levels.

Another class of HETEs, ω-terminal HETEs, are generated from AA by cytochrome P450 (CYP) ω-hydroxylases, primarily CYP4A and CYP4F subfamilies [1]. The two ω-terminal HETEs, 19- and 20-HETEs, are potent oxylipins typically found at very low levels and are notoriously difficult to assess by LCMS. These ω-terminal HETEs were below detectable levels in our samples in both Tie2-CYP2J2 Tr and WT mice. However, since 20-HETE is a potent vasoconstrictor [1], and is involved with the renin-angiotensin system to promote hypertension, vasoconstriction, and vascular dysfunction [47, 48], we evaluated the effect of inhibiting ω-hydroxylases by DDMS. DDMS enhanced CRH in both Tie2-CYP2J2 Tr and WT mice. Evaluating the effect of DDMS on the levels of ω-terminal HETEs would better link this reported functional finding to biochemical changes in these oxylipins.

Another group of AA metabolites of the cyclooxygenase pathway is prostanoids, which include prostaglandins (PGs) and TxB_2_. Endothelial overexpression of CYP2J2 did not cause significant changes in the measured prostanoid levels in Tie2-CYP2J2 Tr compared to WT mice. PGs are generally pro-inflammatory [49], but PG-D_2_ and PG-E_2_ have anti-inflammatory effects as well by secreting the anti-inflammatory IL-10 [49, 50]. We previously published that by targeting the breakdown pathway of EETs (through genetic deletion or pharmacologic inhibition of sEH) the levels of prostanoids were decreased in isolated mouse heart perfusates [17]. Overexpressing the EETs-generating CYP2J2 in this study did not significant change prostanoid levels. Although the two approaches increase EETs levels (increasing their generation or decreasing their breakdown), the different effects on prostanoids suggests other, yet undisclosed, effects on the cyclooxygenase pathway. Interestingly, the levels of PG-F_2α_ and PG-E_2_ were decreased in response to ischemia in both Tie2-CYP2J2 Tr and WT mice. TxB_2_ is the inactive degradation product of TxA_2_ [49], Hellmann et al. suggested that prostaglandins are not involved in post-occlusive reactive hyperemia in the skin [51]. Based on these data, we speculate that prostanoids were not involved in mediating CRH in either mouse genotype.

In addition to arachidonic acid-derived oxylipins, we analyzed linoleic acid-derived oxylipins, EpOMEs and HODEs. EpOMEs are linoleic-acid-derived epoxides, akin to EETs, while the HODEs are hydroxyls similar to mid-chain HETEs. Both isomers of EpOMEs (9,10- and 12,13-EpOMEs) were increased in Tie2-CYP2J2 Tr mice compared to WT. However, their corresponding metabolites, 9,10- and 12,13-DiHOMEs, were not different between the two genotypes. This finding supports previous reports that CYP2J2 has predominantly epoxygenase activity toward LA, to catalyze the formation of EpOMEs [21]. Physiological levels of EpOMEs protected against hypoxia/reoxygenation injury [20, 22], whereas their hydrated metabolites, DiHOMEs, may have been shown to be less active or cytotoxic [7, 20, 27]. The changes in oxylipin levels and CRH in Tie2-CYP2J2 Tr hearts were similar to those seen in sEH^–/–^[11] or sEH inhibitor-treated mice [17]. Oxylipin levels confirm that Tie2-CYP2J2 Tr hearts increase overall oxylipin production, while sEH^–/–^hearts have altered epoxide hydrolysis. sEH^–/–^hearts produce nearly triple the amount 14,15-EET as WTs, with substantially reduced 14,15-DHET formed [11]. Tie2-CYP2J2 Tr hearts produce approximately 50% more 14,15- and 11,12-EET than WT hearts; 14,15- and 11,12-DHET values were also higher than WT, though this was not statistically significant. Similarly, compared to WT hearts, sEH^–/–^hearts produce nearly double the amount 9,10- and 12,13-EpOME-and lower levels of corresponding DiHOMEs [11]. Tie2-CYP2J2 Tr hearts appear to increase both EpOME and DiHOME production. Both sEH^–/–^and Tie2-CYP2J2 Tr hearts display 30% higher CRH than littermate controls. The changes in CRH best correlate with increases in EET levels, either through enhanced production or diminished hyrdrolysis.

In addition to EpOMEs, HODEs can be formed from linoleic acid (LA) through hydroxylation by CYP epoxygenases or lipoxygenases [41]. The two HODE isomers, 9-, and 13-HODE, were not significantly different between Tie2-CYP2J2 Tr and WT. This is consistent with what Moran et al reported: CYP2J2 has predominantly LA-epoxygenase (EpOMEs-forming), but not LA-hydroxylase (HODEs-forming) activity [21]. The physiologic functions of HODEs are not widely investigated [41]. 9- and 13-HODE isomers seem to have opposite effects: 13-HODE may be anti-inflammatory [52–56], whereas 9-HODE could be pro-inflammatory [57, 58]. In this study, 9-, and 13-HODEs were decreased in response to ischemia in both mouse genotypes. We did not observe this response to ischemia by HODEs in previously published papers with similar settings [17]. More investigation is needed to better characterize the role these metabolites may have in modulating the response to ischemia and their response to it. Since endothelial overexpression of CYP2J2 did not change the levels of 9-, and 13-HODEs, we expect that these metabolites did not play a role in the enhanced CRH in Tie2-CYP2J2 Tr compared to WT mice.

8-iso-PGF_2α_, was detected in the heart perfusate and may provide more insight into the mechanism(s) by which CYP2J2 overexpression enhanced CRH in Tie2-CYP2J2 Tr mice. 8-iso-PGF_2α_ was lower in Tie2-CYP2J2 Tr mice. 8-iso-PGF_2α_ is one of the isoprostanes, which are produced by free radical-induced lipid peroxidation of AA [59]. Thus, 8-iso-PGF_2α_, produced under conditions of elevated reactive oxygen species (ROS), serves as a surrogate marker for ROS production [7]. 8-iso-PGF_2α_ is also a potent coronary vasoconstrictor in isolated guinea pig hearts [59]. The level of 8-iso-PGF_2α_ was not affected by ischemia in either mouse genotype possibly because of the short duration of ischemia, but it was reproducibly lower in CYP2J2 overexpressed mice pre- and post- ischemia. This decrease in 8-iso-PGF_2α_, and subsequent decrease in vasoconstrictive activity, may have contributed to the enhanced CRH observed in Tie2-CYP2J2 Tr mice.

In summary, the findings of this study demonstrate that endothelial overexpression of CYP2J2 enhances CRH possibly through augmenting the CYP epoxygenase pathway, which was manifested by increased EETs, increased EpOMEs, and decreased 8-iso-PGF_2α_. The effects of CYP2J2 overexpression on these pathways might have collectively accounted for the observed increase in CRH. Also, our findings demonstrate that inhibition of the CYP epoxygenase pathway attenuated, whereas inhibition of ω-hydroxylases enhanced CRH. Additionally, short ischemia caused decrease in mid-chain HETEs and HODEs. Neither CYP2J2 overexpression nor ischemia produced changes in DHETs, DiHOMEs, and prostanoids. Therefore, we conclude that CYP2J2 overexpression and inhibition of ω-hydroxylases enhance, whereas inhibition of CYP epoxygenase pathway attenuates CRH.
